# Does living near a tobacco retailer impact the efficacy of smoking cessation treatments?: Analysis from a randomized trial

**DOI:** 10.1016/j.abrep.2025.100635

**Published:** 2025-10-18

**Authors:** Margarita Santiago-Torres, Kristin E. Mull, Brianna M. Sullivan, Jonathan B. Bricker

**Affiliations:** a Fred Hutchinson Cancer Center, Division of Public Health Sciences, Seattle, WA, USA; b University of Washington, Department of Psychology, Seattle, WA, USA

**Keywords:** Acceptance & Commitment Therapy, Digital interventions, iCanQuit, QuitGuide, Smartphone applications, Smoking cessation, Tobacco retailer density

## Abstract

**Introduction:**

Whether living near a tobacco retailer impacts the efficacy of smoking cessation treatments remains largely unknown. We used data from a randomized trial comparing two smoking cessation apps among 2415 adults: iCanQuit, based on Acceptance and Commitment Therapy, and QuitGuide, based on standard U.S. Clinical Practice Guidelines. We examined whether tobacco retailer density impacted the efficacy of the treatments on 12-month cessation outcomes.

**Methods:**

Data on tobacco retailer density per 1,000 people (i.e., “one unit”) was linked to each participant’s residential ZIP Code. Cessation outcomes included 30-day point prevalence abstinence (PPA) from cigarette smoking, prolonged abstinence, 30-day PPA from nicotine/tobacco products, and relapse. We examined the interaction between density and treatment arm on 12-month 30-day PPA and compared cessation outcomes separately by arm.

**Results:**

The interaction between density and treatment arm on cessation did not reach statistical significance (*P* = 0.09). For each one-unit increase in density, there was no change in quit rates in the iCanQuit arm (*P* = 0.62). In the QuitGuide arm, higher density was associated with lower quit rates (OR = 0.54; 95 % CI, 0.27–1.06; *P* = 0.07), although not-statistically significant. There was a significant interaction between density and treatment arm on prolonged cigarette abstinence (*P* = 0.03). We found no change in prolonged abstinence in the iCanQuit arm (*P* = 0.44). In the QuitGuide arm, higher density was associated with lower prolonged abstinence (OR = 0.27; 95 % CI, 0.07–1.02; *P* = 0.054), although not-statistically significant. *Conclusions:* Living near tobacco retailers may undermine the effectiveness of standard behavioral treatment but appears to have no impact on the effectiveness of acceptance-based smoking cessation treatments.

## Highlights

• Tobacco retailer density had no effect on the acceptance-based iCanQuit treatment.
• Retailer density lowered prolonged abstinence in USCPG-based QuitGuide treatment.
• Acceptance-based cessation treatments should be made more available.

## 1 Introduction

While progress has been made in reducing cigarette smoking in the United States (U.S.), major geographic disparities in smoking persist, including among individuals who live in communities with a high density of tobacco retailers (Center for Disease Control and Prevention, 2021, U.S. Department of Health and Human Services., 2020). Population-based research shows that greater accessibility of tobacco products is associated with higher smoking prevalence, lower likelihood of cessation, and increased relapse among individuals trying to quit (Finan et al., 2019, Golden et al., 2020, Marsh et al., 2021, Nuyts et al., 2021, Valiente et al., 2021). The accumulating evidence suggests that even if individuals have access to cessation treatments, a major barrier to quitting success is their proximity to tobacco retailers.

Individuals in communities with a high density of tobacco retailers are also especially vulnerable to relapse due to greater exposure to tobacco advertisements and visual cues prompting smoking (Kong et al., 2021, Loomis et al., 2012, Pearce et al., 2009). Higher exposure to tobacco marketing at the point of sale creates significant barriers to quitting success (Herd et al., 2009). Furthermore, abstaining from cigarette smoking is particularly difficult for individuals with high nicotine dependence, as they experience strong physical and psychological barriers to quit (Santiago-Torres et al., 2023). Tobacco advertisements and visual cues that lead to cravings to smoke are a strong driver of relapse, making it crucial to identify treatments that directly address these cravings.

One such treatment is Acceptance and Commitment Therapy (ACT) (Hayes et al., 2013). In ACT, acceptance means making room for cravings that trigger smoking while allowing them to come and go. The goal of ACT-based strategies for smoking cessation focuses on becoming aware of cravings to smoke and being willing to allow them without smoking (Martinez-Vispo et al., 2020, Potter et al., 2021). These components distinguish ACT from standard behavioral treatments such as U.S. clinical practice guidelines (USCPG)-based smoking cessation programs that focus on avoiding cravings to smoke (Fiore et al., 2008). This distinction is important because avoiding cravings may be impractical for individuals who are frequently exposed to tobacco retailers.

ACT is an evidence-based approach that has demonstrated efficacy in helping adults quit cigarette smoking via acceptance of cravings to smoke (Bricker et al., 2014, Bricker et al., 2021, Karekla and Savvides, 2021, Mak et al., 2020, Mak et al., 2021, McCallion and Zvolensky, 2015, Santiago-Torres et al., 2021, Vilardaga et al., 2019). ACT-based interventions for smoking cessation could help individuals living in communities with high density of tobacco retailers effectively cope with these external factors, because they focus on increasing one’s ability to recognize and be open to experiencing discomfort associated with cravings without acting on them.

Our team developed iCanQuit (citations removed for anonymized review), an ACT-based smartphone application (app) for smoking cessation. In a two-arm randomized controlled trial (RCT), the efficacy of iCanQuit was tested against a USCPG-based app (QuitGuide) among 2415 adults who smoke (citations removed for anonymized review). Compared to QuitGuide, iCanQuit was 1.49 times more efficacious (OR = 1.49; 95 % CI: 1.22–1.83) in helping adults quit cigarette smoking by 12-months (citations removed for anonymized review). The effect of the intervention on quitting cigarette smoking was mediated by changes in ACT core processes, specifically, acceptance of cravings that cue smoking (citations removed for anonymized review). Unknown is whether living near tobacco retailers impacts the effectiveness of behavioral smoking cessation treatments, particularly within the context of an RCT, which would allow for examining how tobacco retailer density may modify treatment effectiveness under controlled conditions.

To help address this major gap, this analysis utilized data from the iCanQuit parent RCT, to explore whether community-level tobacco retailer density impacted the efficacy of an individual-level smoking cessation program. We hypothesized that participants living in areas with higher retailer density would have lower odds of quitting smoking at 12 months compared to those in areas with lower retailer density. This hypothesis is based on observational population-based data showing that high retailer density is associated with reduced cessation success (Finan et al., 2019, Golden et al., 2020, Marsh et al., 2021, Nuyts et al., 2021, Valiente et al., 2021). This finding would be refuted if no statically significant association was observed or if the direction of the effect was opposite to that hypothesized.

Because the parent RCT compared two distinct behavioral cessation interventions, we first investigated whether tobacco retailer density moderated the treatment effect on cessation outcomes. This step was important to avoid conflating environmental effects with differences between interventions. Conceptually, iCanQuit teaches users skills to recognize and accept cravings without acting on them, whereas QuitGuide emphasizes avoiding cravings—a strategy that may be impractical for individuals living near tobacco retailers. We then evaluated the association between retailer density and cessation *separately within each treatment arm*. Results will inform tailoring of cessation programs for individuals based on their environmental context.

## 2 Methods

### 2.1 Study overview

The details of the iCanQuit parent trial have been previously published, including the study protocol as supplemental material (citations removed for anonymized review). The parent trial involved human participants and study procedures were approved by the Fred Hutchinson Cancer Center Institutional Review Board (IRB protocol number: FHIRB0008317) and registered at Clinicaltrials.gov (number removed for anonymized review). Participants gave informed consent to participate in the study.

### 2.2 Participants

Adults who smoked five cigarettes per day or more, wanted to quit smoking within the next 30 days, and if concurrently using any other tobacco or nicotine products (e.g., e-cigarettes), wanted to quit using them within the next 30 days. Additional eligibility criteria included access to their own smartphone, not currently participating in other smoking cessation interventions, no prior participation in our studies, and no household or family member participating.

### 2.3 Recruitment and enrollment

Participants were recruited primarily via social media from May 27, 2017, to September 28, 2018. Those who screened eligible and provided their email received an instant email invitation to complete a secured online survey for informed consent and baseline survey. To deter fraud we used, (1) CAPTCHA authentication, (2) checked IP addresses for suspicious activity, and when needed, (3) staff verified eligibility and contact details via phone. Interested and eligible participants were randomly assigned 1:1 to either iCanQuit or QuitGuide using randomly permuted blocks of size 2, 4, and 6, stratified by daily smoking frequency (≤20 vs. ≥ 21 cigarettes per day), educational level (high school or less vs. some college or more), race/ethnicity (minority race/ethnicity vs non-Hispanic White), and positive screen for experiencing depression symptoms (Center for Epidemiological Studies Depression Scale, CES-D 16 score ≤ 15 vs. ≥ 16) (Radloff, 1977).

### 2.4 Interventions

#### 2.4.1 iCanQuit

iCanQuit focuses on teaching ACT skills for managing smoking cravings, motivation to quit, and relapse prevention (citations removed for anonymized review). Users create personalized quit plans and navigate through eight content levels. Users receive on-demand help in coping with smoking cravings, track daily cigarette consumption, and monitor cravings passed without smoking. An avatar “guide” aids navigation through the app and teaches ACT skills for handling cravings. If the user lapses (e.g., records smoking a cigarette), the program encourages them to set a new quit date and return to the section for preparation. iCanQuit targets two ACT-based processes: acceptance and values. The acceptance component teaches skills to accept cravings that cue smoking via distancing, mindfulness skills, and perspective-taking. The values component helps users enact personal values (e.g., health, family, personal goals) and what matters most to them as motivators to quit.

#### 2.4.2 QuitGuide

QuitGuide follows the USCPG for smoking cessation and focuses on increasing motivation to quit by using reason and logic and providing information on the health consequences of smoking (Fiore et al., 2008). The application helps users develop a quit plan, identify smoking behaviors, triggers, and reasons for being smoke-free, and identify sources of social support for quitting. It teaches skills for avoiding situations that lead to cravings to smoke, staying smoke-free, and coping with slips. Main components are as follows: (1) “Thinking about Quitting”, which encourages users to think of motivations for quitting and provides information on the general health consequences of smoking and quitting; (2) “Preparing to Quit”, which helps users develop a quit plan, identify smoking behaviors, triggers, and reasons for being smoke-free, identify social support for quitting, and provides information on FDA-approved cessation medications; (3) “Quitting”, which teaches skills for avoiding cravings to smoke, such as finding replacement behaviors (e.g., chewing on carrot sticks) and staying busy; and (4) “Staying Quit”, which has tips, motivations, and actions to stay smoke-free and skills for coping with slips via fighting cravings and trying to be positive.

While neither the ACT-based iCanQuit app *nor* the USCPG-based QuitGuide app directly addresses external factors such as high density of tobacco retailers, the teaching of acceptance of cravings in ACT-based smoking cessation interventions is conceptually distinct from existing USCPG-based approaches that teach avoidance of cravings. This distinction is relevant here because the avoidance of cravings may be impractical in communities with high density of tobacco retailers. Therefore, ACT-based interventions for smoking cessation could help individuals residing in areas with high density of tobacco retailers to more effectively cope with these external factors because they focus on increasing one’s ability to recognize and be open to experiencing discomfort associated with cravings without acting on them.

Participants received access to the apps for a period of 12 months. It was anticipated that participants would finish the intervention content in a period of six to eight weeks post-randomization. Over the course of 12 months, the median length of application use post-randomization was 22 days for iCanQuit vs. 23 days for QuitGuide participants. This difference was not statistically significant. With regards to acceptability and adherence to treatment, they were high in both treatment arms, although, these were significantly higher for iCanQuit compared to QuitGuide (e.g., number of times the application was used, iCanQuit 37.5 vs. 9.9 QuitGuide, *P* = 0.001; overall satisfaction with the assigned app, iCanQuit 88.5 % vs. 77.1 % QuitGuide, *P* = 0.001). These results have been previously reported elsewhere (Bricker et al., 2023, Bricker et al., 2021, Bricker et al., 2022, Bricker et al., 2020).

### 2.5 Measures

#### 2.5.1 Baseline characteristics

At baseline participants reported on sociodemographic characteristics, including age, gender identity, race and ethnicity, socioeconomic status (education, income, employment), smoking behaviors (i.e., cigarette dependence) (Heatherton et al., 1991), depression via the Center for Epidemiological Studies Depression Scale (Radloff, 1977), and residential ZIP Code as well as smoking behaviors via online self-administered questionnaires.

#### 2.5.2 Tobacco retailer density

To assess tobacco retailer density, we used the National Neighborhood Data Archive (NaNDA) data on (1) tobacco stores and stands, and (2) convenience stores to link measures of tobacco retailer density to each participant’s residential ZIP Code Tabulation Area (ZCTA) (Melendez et al., 2024, Research, 2025). Because not all ZIP Codes are the same as their ZIP Code Tabulation Area, participant ZIP Codes were linked to ZCTAs using standard ZIP-to-ZCTA crosswalk data (U.S. Department of Health and Human Services, 2024), which follows U.S. Census Bureau guidance for approximating ZIP Codes with areal units and the R library ‘zippeR’ (Prener et al., 2025). This approach is widely applied in neighborhood effects research and has been previously validated via geocoding and data cleaning procedures in the development of NaNDA datasets (Finlay et al., 2019, Hirsch et al., 2021, Kaufman et al., 2015). Tobacco stores and stands are defined as establishments primarily engaged in the retail sale of cigarettes, cigars, tobacco, and smokers' supplies.

Convenience stores are defined as establishments primarily engaged in retailing a limited line of groceries and includes gasoline service stations with convenience stores. Because convenience stores also commonly sell cigarettes and tobacco products (Golden et al., 2022), we considered both tobacco stores and stands and convenience stores to be tobacco retailers. The primary tobacco retailer density measure was the unit of per capita density, i.e., the number of tobacco retailers per 1,000 people, with higher values indicating more establishments per person. Tobacco retailer density by number of retailers per 1,000 people was selected as our primary exposure variable to account for the relative availability of outlets within the population, rather than only their geographic concentration. This per capita measure has been shown in prior systematic reviews to be also more consistently associated with smoking prevalence, initiation, and cessation outcomes than area-based or proximity measures (Marsh et al., 2021, Valiente et al., 2021).

#### 2.5.3 Smoking cessation

The primary smoking cessation outcome was self-reported, complete-case 30-day point prevalence abstinence (PPA) from cigarette smoking, or no smoking at all in the past 30 days, at the 12-month follow-up. Secondary smoking cessation outcomes included missing-as-smoking and multiple imputation of the primary cessation outcome (30-day PPA from cigarette smoking). Missing-as-smoking analysis is also referred to as an intention-to-treat approach, which includes all participants in the study and counts them as smoking if their smoking status at the follow-up timepoint is unknown (West et al., 2005). Additional outcomes included prolonged abstinence (defined as no smoking since 3-months post-randomization, based on participant’s self-reported date of last cigarette) and cessation of all nicotine and tobacco products (e-cigarettes or vaping, chewing tobacco, snus, hookahs, cigars, cigarillos, tobacco pipes, and kreteks). We also assessed relapse at 12 months, which was defined as reporting no smoking in the past 30 days at the 6-month follow-up but reporting smoking in the past 30 days at the 12-month follow up.

#### 2.5.4 Acceptance of cravings to smoke

Acceptance of internal cues to smoke was measured using the 27-item Avoidance and Inflexibility Scale (AIS-27) adapted from Gifford et al. (Gifford et al., 2004), a validated tool that includes three subscales assessing willingness to experience smoking-related physical sensations, emotions, and thoughts that cue smoking (Farris et al., 2015). Items are rated on a 5-point scale from (1) “Not at all” to (5) “Very willing” and averaged, with higher scores indicating greater acceptance”. The AIS was not designed to measure environmental cues. A sample physical sensation item was “How willing are you to notice these bodily sensations without smoking?” and items from the emotions and thoughts subscales were similar, substituting “feelings” or “thoughts” for “bodily sensations. Acceptance of internal cues to smoking was found to mediate the treatment effect of iCanQuit on smoking cessation outcomes in the parent RCT, providing evidence supporting this process-based mechanism of action (Bricker et al., 2021).

### 2.6 Statistical analysis

For this analysis, we included all but one participant (n = 2414/2415, >99.9 %) whose residential ZIP Codes could be tied to ZCTA. We used ZCTA and NaNDA data specific to the year of randomization (i.e., 2017 or 2018) for each participant. To test whether the odds of quitting and relapse are associated with tobacco retailer density, we used logistic regression models with an interaction between treatment arm and density. Subsequently, we investigated the association between tobacco retailer density and cessation or relapse separately in each treatment arm. The R library ‘mice’ (van Buuren S. & K., 2011) was used for multiple imputation of missing cessation outcome data, creating 10 complete data sets and pooling model results (D.B., 1987). Multiple imputation was used to assess the sensitivity of the results to assumptions around missing data for the 30-day PPA cessation outcome (van Buuren S. & K., 2011), a widely used method to perform multiple imputation of missing data with fully conditional specification, in which missing smoking outcome is imputed conditional on all other variables in the imputation model (van Buuren S. & K., 2011).

Finally, in post-hoc analyses we used linear regression separately in each treatment arm to test whether tobacco retailer density was associated with changes in acceptance of cravings from baseline to the 3-month follow-up. These models were adjusted for baseline acceptance scores. All cessation models were also adjusted for factors used in stratified randomization to avoid losing power and obtaining incorrect 95 % CI (Kernan et al., 1999), including, daily smoking frequency, education, minority race/ethnicity background, and positive screening for depression via the Center for Epidemiological Studies Depression Scale (Radloff, 1977). We also adjusted for baseline characteristics that both differed between treatment arms and were associated with the primary cessation outcome, to reduce the potential for confounding as indicated in Table footnotes (Assmann et al., 2000, Kernan et al., 1999). All statistical tests were 2-sided, with α = 0.05.

## 3 Results

### 3.1 Participant characteristics

Participants were from all 50 U.S. states with approximately 22.8 % living in a rural area as defined by Rural-Urban Commuting Area (RUCA) codes that classify U.S. Census tracts based on population density, urbanization, and daily commuting. (US Department of Agriculture, Economic Research Service, 2020. URL:https://www.ers.usda.gov/data-products/rural–urban-commuting-area-codes/ [accessed 2021–02-21].). Baseline characteristics for adults who enrolled in the iCanQuit parent randomized trial have been previously reported (citations removed for anonymized review). Briefly, outcome data retention at 12 months was high (87.2 %), and baseline characteristics were balanced between the two treatment arms. On average, participants were 38.2 (10.9) years old, 70.4 % female, 35.9 % were from racial or ethnic minority groups (non-White or Hispanic), and 41.2 % had lower education (high school diploma or less). Regarding smoking, most (83.2 %) had smoked for 10 years or longer, 74.7 % smoked 11 cigarettes or more per day, and 60.1 % had high cigarette dependence (defined as Fagerström Test for Cigarette Dependence, FTCD score of 6 or higher) (Heatherton et al., 1991). About 23.8 % reported using e-cigarettes and 38.7 % reported making at least one quit attempt in the past 12 months.

### 3.2 Tobacco retailer density

Data linked to each participant’s residential ZCTA revealed that on average, the density of tobacco retailers was 0.32 (SD = 0.23) per 1,000 people (median = 0.29; range = 0.00–3.82), and the total number of tobacco retailers in each participant’s residential ZCTA was 8.56 (SD = 6.62) and median = 7 (range = 0–38).

### 3.3 Moderation effect of retailer density on smoking cessation at 12 months

There was no interaction between tobacco retailer density and treatment arm on 30-day PPA from cigarette smoking for complete-case [β (SE) 0.84 (0.49), *P* = 0.09; Table 1)] and missing-as-smoking [β (SE) 0.85 (0.48), *P* = 0.09)] nor multiple imputation [β (SE) 0.77 (0.49), *P* = 0.11)]. There was a statistically significant interaction between tobacco retailer density and treatment arm on prolonged cigarette abstinence (*P* = 0.03) indicating that the association was moderated by treatment arm assignment (iCanQuit vs. QuitGuide). We found no interaction between tobacco retailer density and treatment arm on 30-day PPA from nicotine and tobacco products (*P* = 0.21) nor on relapse (*P* = 0.81) at 12 months.

**Table 1:** Tobacco retailer density and smoking cessation and relapse at 12 months.

Cessation outcomes^a^	Interaction between retailer density and treatment arm β (SE), *P* value	Treatment Arm	N	Association between retailer density and cessation outcome OR (95 % CI)	*P* value
30-d PPA from cigarette smoking^b^					
Complete case	0.84 (0.49), p = 0.09	iCanQuit	1039	1.19 (0.60, 2.36)	0.62
		QuitGuide	1067	0.54 (0.27, 1.06)	0.07
Missing-as-smoking	0.85 (0.48), p = 0.07	iCanQuit	1213	1.28 (0.66, 2.48)	0.47
		QuitGuide	1201	0.56 (0.29, 1.09)	0.09
Multiple imputation	0.77 (0.49), p = 0.11	iCanQuit	12,130	1.15 (0.59, 2.25)	0.67
		QuitGuide	12,010	0.55 (0.28, 1.08)	0.08
Prolonged cigarette abstinence^c^	1.76 (0.83), p = 0.03	iCanQuit	839	1.46 (0.56, 3.76)	0.44
		QuitGuide	871	0.27 (0.07, 1.02)	0.054
30-d PPA from nicotine and tobacco products	0.65 (0.52), p = 0.21	iCanQuit	1038	1.15 (0.56, 2.37)	0.70
		QuitGuide	1068	0.63 (0.30, 1.30)	0.21
Relapse between 6 months and 12 months^d^	−0.30 (1.25), p = 0.81	iCanQuit	248	0.62 (0.13, 2.99)	0.55
		QuitGuide	149	0.71 (0.11, 4.59)	0.72

Abbreviations: OR, odds ratio; PPA, point prevalence abstinence; SE, standard error.

a All models were adjusted for factors used in stratified randomization including positive screening for depression, daily smoking frequency, education, minority race/ethnicity backgrounds.

b Models for 30-day PPA from cigarette smoking and 30-day PPA from all nicotine and tobacco products are also adjusted for gender, which was identified as a potential confounder.

c Prolonged abstinence is defined as no smoking since 3-months post-randomization, using self-reported date of last cigarette.

d Relapse is defined as reported abstinence at the 6-month follow-up, followed by report of cigarette smoking at the 12-month follow-up.

### 3.4 Tobacco retailer density and smoking cessation by treatment arm

At 12 months, we found that for every one unit increase in retailer density, there was no evidence of a difference in the odds of quitting cigarette smoking (30-day PPA, complete-case; Table 1) in the iCanQuit arm (OR = 1.19, 95 % CI, 0.60, 2.36, *P* = 0.62), but there was a trend towards a reduction in the odds of quitting in the QuitGuide arm (OR = 0.54, 95 % CI, 0.27, 1.06, *P* = 0.07). Similar effect sizes were observed for 30-day PPA missing-as-smoking [iCanQuit: 1.28 (0.66, 2.48); QuitGuide: 0.56 (0.29, 1.09)] and multiple imputation outcomes [iCanQuit: 1.15 (0.59, 2.25); QuitGuide: 0.55 (0.28, 1.08)]. There was no evidence of an association between tobacco retailer density and prolonged abstinence from 3 to 12 months in the iCanQuit arm (OR = 1.46, 95 % CI, 0.56, 3.76, *P* = 0.43), but there was a trend that did not reach statistical significance for a 73 % decrease in prolonged abstinence in the QuitGuide arm (OR = 0.27, 95 % CI, 0.07, 1.02, *P* = 0.054). There was no evidence in either treatment arm of an association between tobacco retailer density and 30-day PPA from all nicotine tobacco products outcomes [iCanQuit: 1.15 (0.56, 2.37); QuitGuide: 0.63 (0.30, 1.30)] or relapse outcomes [iCanQuit: 0.62 (0.13, 2.99); QuitGuide: 0.71 (0.11, 4.59)] at 12 months.

### 3.5 Association between retailer density and acceptance of internal cues to smoke

In post-hoc analysis, we explored whether there was an association between tobacco retailer density and change from baseline to 3 months in acceptance of internal cues to smoke, as measured by acceptance of (1) physical sensations, (2) emotions, and (3) thoughts that may cue smoking and by the mean of these three subscales (Table 2). There was a non-statistically significant trend for an association between retailer density and change in acceptance of emotions that cue smoking in the QuitGuide arm (Point Estimate, PE: −0.12, 95 % CI, −0.25, 0.01, *P* = 0.06) such that individuals in the QuitGuide arm living in areas with higher retailer density had decreases in acceptance of emotions that cue smoking from baseline to 3 months. Whereas there was no evidence of an association in the iCanQuit arm (PE: 0.05, 95 % CI, −0.15, 0.25, *P* = 0.63). No other significant associations were observed between retailer density and acceptance scores.

**Table 2:** Tobacco retailer density prediction of change in acceptance of internal cues to smoke.

Variable^a^, ^b^	Treatment Arm	N	Change from baseline to 3-months Mean (SD)	Association between retailer density and change in acceptance Point Estimate (95 % CI)	*P* value
Acceptance of internal cues to smoke					
Physical sensations	iCanQuit	995	0.22 (0.77)	−0.03 (−0.23, 0.18)	0.80
	QuitGuide	999	0.09 (0.68)	−0.02 (−0.16, 0.13)	0.84
Emotions^c^	iCanQuit	1000	0.20 (0.71)	0.05 (−0.15, 0.25)	0.63
	QuitGuide	1023	0.05 (0.60)	−0.12 (−0.25, 0.01)	0.06
Thoughts^d^	iCanQuit	1003	0.19 (0.66)	0.07 (−0.11, 0.25)	0.44
	QuitGuide	1024	0.05 (0.56)	−0.07 (−0.19, 0.05)	0.23
Acceptance mean score^d^	iCanQuit	986	0.20 (0.62)	0.04 (−0.14, 0.22)	0.65
	QuitGuide	994	0.06 (0.50)	−0.05 (−0.16, 0.06)	0.36

Abbreviations: SD, standard deviation.

a All models were adjusted for factors used in stratified randomization including positive screening for depression, daily smoking frequency, education, minority race/ethnicity backgrounds.

b All changes in acceptance scores calculated as value at 3-months minus baseline value.

c Avoidance and Inflexibility Scale. Range in change is − 4 to 4. Positive scores indicate higher acceptance at follow-up assessments.

d Models for acceptance of emotions, thoughts, and mean are also adjusted for the potential confounder gender.

## 4 Discussion

We found that living near tobacco retailers may moderate the efficacy of individual-level smoking cessation interventions on long-term smoking cessation outcomes. The impact of tobacco retailer density had no effect on smoking cessation among participants who received the ACT-based iCanQuit app intervention. Although not statistically significant (*P* = 0.07), the observed 46 % reduction in the odds of quitting cigarette smoking at 12 months (30-day point prevalence abstinence) in the QuitGuide arm suggest a potential moderation role of tobacco retailer density that warrants further study. Additionally, there was a significant interaction between tobacco retailer density and treatment arm on prolonged abstinence or reporting no cigarette smoking since 3-months post-randomization, (*P* = 0.03). We found no effect of retailer density on prolonged abstinence among participants who received the ACT-based intervention (iCanQuit), but and although not statistically significant (*P* = 0.06), we also observed a 73 % reduction in the odds of prolonged abstinence in the QuitGuide arm.

We attribute these findings to the fact that the ACT theory-based smoking cessation treatment (iCanQuit) teaches individuals skills to be willing and open to experience cravings even while exposed to tobacco retailers. Although not statistically significant (*P* = 0.06), post-hoc analyses suggested a potential association between higher tobacco retailer density and lower acceptance of emotions that cue smoking in the QuitGuide arm, that was *not observed* in the iCanQuit arm. This exploratory finding warrants further study. These results suggest that skills in acceptance of internal emotional cues to smoke protected iCanQuit participants from exposure to tobacco retailers whereas, the absence of acceptance skills left QuitGuide participants more vulnerable to effects of this exposure. Therefore, the ACT-based core process of acceptance may have broader applicability to environmental cues influencing smoking cessation.

In this sample with over 60 % of participants having high baseline cigarette dependence (FTCD scores of 6 or higher), learning skills to accept sensations, emotions, and thoughts that cue smoking via distancing, mindfulness skills, and perspective-taking seems critical in sustaining abstinence when living near tobacco retailers. In contrast, the USCPG-based approach that promotes avoiding places and situations known to induce cravings to smoke may not be an effective approach for quitting smoking for those who live near tobacco retailers. These findings suggest ACT techniques like acceptance can help people who are trying to quit smoking by reducing the impact of environmental triggers.

A prior study of a randomized controlled trial (RCT) of a text messaging smoking cessation intervention among 197 African American/Black urban adolescents evaluated the effects of tobacco retailer density on *momentary smoking* (not smoking cessation) at 2, 4, and 6 months (Mennis et al., 2016). Although, the smoking outcomes of this trial are not directly comparable to the current study, the findings similarly demonstrated that tobacco retailer density influences smoking behavior within the context of an RCT. The study also found that the intention to smoke was associated with retailer density, in that individuals living in neighborhoods with a higher density of tobacco retailers were more likely to smoke more frequently than their counterparts. Notably, the relationship between retailer density and smoking was significantly stronger in the control condition than in the experimental arm, suggesting that the efficacy of an intervention is dependent, to some degree, on environmental factors, such as the density of nearby tobacco retailers.

## 5 Strengths and limitations

The current study has important strengths. To our knowledge, *this is one of few studies* to examine the impact of tobacco retailer density on response to a smoking cessation treatment within the context of a randomized controlled trial. The study therefore helps address gaps into knowledge about the role of tobacco retailer proximity in smoking cessation that could eventually inform the development of future interventions. Second, the study included a large national sample of 2415 adults from all 50 U.S. states, a geographically and racially diverse representative sample, with 22.8 % of participants residing in rural areas and 35.9 % racial/ethnic minorities (citations removed for anonymized review). Third, the data included long-term cessation outcomes, which allowed for evaluating prolonged abstinence and relapse by the 12-month follow-up (Kocak et al., 2015, Walker et al., 2006).

There are important limitations of our study. First, because it would be impossible to randomly assign people to their residences, the necessity of only looking at associations is an inherent limitation to answering the question of whether tobacco retailer density impacts cessation treatment efficacy. Second, smoking cessation outcomes were self-reported, which introduces potential reporting bias, although this bias would not be expected to differ between treatment arms. Third, population-based data on tobacco retailers was linked to participants’ residential ZIP Code and not to other settings such as workplaces. Finally, the study did not assess participants’ actual engagement with tobacco retailers, therefore real-world research experiments are needed to provide additional insights into the environmental influences on cessation when exposed to tobacco retailers.

## 6 Clinical implications

There are also important implications of our findings. With regards to individual-based treatments, our results show how greater exposure to tobacco retailers can undermine quit attempts, especially among individuals receiving USCPG-based treatments. The results suggest these standard treatments may not be effective in environments that promote smoking. Behavioral treatments for smoking cessation which focus on acceptance of cravings should be made more available—especially to individuals living near tobacco retailers. Acceptance-based treatments teach the individual “to control what you can and accept what you can’t”. In smoking-promoting environments, cue-induced cravings are inevitable during quitting attempts. Therefore, learning to manage cravings with acceptance rather than avoidance may be the key to quitting success.

Given the marginal increased risk of relapse found in the control condition as tobacco retailer density increases, a potential solution could be increased regulation of tobacco retailers. Our results provide evidence that access to tobacco retailers in residential areas should be regulated as part of tobacco control strategies (Philbin et al., 2025, Trangenstein et al., 2025, White et al., 2025). While certain tobacco control policies, such as price increases, media campaigns, and smoke-free policies have been shown to contribute to the overall decrease in cigarette smoking in the U.S., tobacco retailers continue to be the prominent driver of access to and marketing of tobacco products (Kong & King, 2021). Retailer-focused initiatives, such as licensing caps and zoning restrictions, can serve as an important complement to effective smoking cessation treatments at the individual level, becoming more impactful when combined.

## 7 Conclusion

Living near tobacco retailers seems to undermine the effectiveness of a standard behavioral smoking cessation treatment but not of an acceptance-based smoking cessation treatment. Smoking cessation treatments based on acceptance of cravings to smoke could be beneficial for those living in areas with a high density of tobacco retailers.

## CRediT authorship contribution statement

**Margarita Santiago-Torres:** Writing – original draft, Conceptualization. **Kristin E. Mull:** Writing – review & editing, Formal analysis, Data curation, Conceptualization. **Brianna M. Sullivan:** Writing – review & editing, Project administration. **Jonathan B. Bricker:** Writing – review & editing, Supervision, Funding acquisition, Conceptualization.

## Declaration of competing interest

The authors declare that they have no known competing financial interests or personal relationships that could have appeared to influence the work reported in this paper. The study was funded by grant R01CA192849 awarded to Jonathan B. Bricker from the National Cancer Institute.

## Data Availability

Data will be made available on request.
